# Multi-marker approach using C-reactive protein, procalcitonin, neutrophil CD64 index for the prognosis of sepsis in intensive care unit: a retrospective cohort study

**DOI:** 10.1186/s12879-022-07650-6

**Published:** 2022-07-30

**Authors:** Na Huang, Jing Chen, Yu Wei, Yongrui Liu, Kang Yuan, Jingli Chen, Mingfeng He, Nan Liu

**Affiliations:** 1 Foshan Hospital of Traditional Chinese Medicine, Foshan, China; 2 Department of Critical Care Medicine, The First Affiliated Hospital of GuangZhou University of Chinese Medicine, Guangzhou, China; 3 Basic Medical College, Guangzhou University of Chinese Medicine, Guangzhou, China; 4 Emergency Department, The First Affiliated Hospital of GuangZhou University of Chinese Medicine, Guangzhou, China; 5 Emergency Department, Foshan Hospital of Traditional Chinese Medicine, Foshan, China

**Keywords:** Sepsis, Prognosis, C-reactive protein, Procalcitonin, Neutrophil CD64 index

## Abstract

**Background:**

We aimed to explore the prognostic utilities of C-reactive protein (CRP), procalcitonin (PCT), neutrophil CD64 (nCD64) index, in combination or alone, in septic patients.

**Methods:**

We retrospectively included 349 septic patients (based on Sepsis 3.0 definition). The primary outcome was 28-day all-cause mortality. Cox regression model, receiver-operating characteristic (ROC) curve, reclassification analysis, Kaplan–Meier survival curves were performed to evaluate the predictive efficacy of the above parameters.

**Results:**

CRP, nCD64 index were independent predictors of 28-day mortality for sepsis in the Cox regression model [CRP, HR 1.004 (95% CI 1.002–1.006), P < 0.001; nCD64 index, HR 1.263 (95% CI 1.187–1.345, P < 0.001]. Area under the ROC curve (AUC) of CRP, PCT, nCD64 index, nCD64 index plus PCT, nCD64 index plus CRP, were 0.798 (95% CI 0.752–0.839), 0.833 (95% CI 0.790–0.871), 0.906 (95% CI 0.870–0.935), 0.910 (95% CI 0.875–0.938), 0.916 (95% CI 0.881–0.943), respectively. nCD64 plus CRP performed best in prediction, discrimination, and reclassification of the 28-day mortality risk in sepsis. The risk of 28-day mortality increased stepwise as the number of data exceeding optimal cut-off values increased.

**Conclusions:**

nCD64 index combined with CRP was superior to CRP, PCT, nCD64 index and nCD64 index plus PCT in predicting 28-day mortality in sepsis. Multi-marker approach could improve the predictive accuracy and be beneficial for septic patients.

## Background

Sepsis is defined as a life-threatening organ dysfunction due to a dysregulated host response to infection [1]. Septic patients may present with both hyperinflammatory and immunosuppressive phenotypes [2]. Worldwide, sepsis remains a major cause of mortality, and early stratification of these critically ill patients helps to decrease mortality and disability [3, 4]. The prognosis of septic patients is mainly based on severity scores and some biomarkers [5]. C-reactive protein (CRP), procalcitonin (PCT), neutrophil CD6 (nCD64) are among the most studied biomarkers, which have shown varying power to predict patient severity in previous studies [2, 6–9]. CRP and PCT, cheap and readily available, are by far the most routinely used biomarkers for sepsis [10]. Both of them, produced as acute-phase reactants in infection, represent the inflammation status of septic individuals. Neutrophils are first-line defence cells of innate immunity responding to the infecting pathogen. On resting neutrophil, CD64 expression is low and it is significantly up-regulated within few hours when activated, making nCD64 a good biomarker for infection and sepsis [11].

Sepsis is highly heterogeneous because of various pathogens and different host responses to inflammation. Clinicians need to quickly stratify the disease progression with simple, fast but useful tests. In the present study, we aimed to assess predictive accuracy of CRP, PCT, neutrophil CD64 index, in combination or alone, in predicting 28-day mortality in sepsis. We hypothesized that multi-marker approach using CRP, PCT, neutrophil CD64 index could be a better strategy than single biomarker assessment for septic patients.

## Methods

### Study design and participant enrollment

**Fig. 1:**
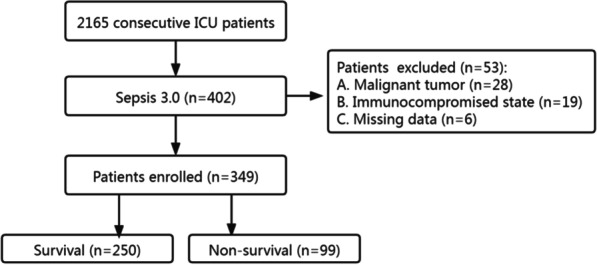
Flowchart of the enrolled patients

This was a single-center retrospective study carried out at the First Affiliated Hospital of GuangZhou University of Chinese Medicine. From January 2018 to July 2021, a total of 402 consecutive septic patients (no COVID-19 patients) were admitted to the Intensive Care Unit (ICU) (Fig. 1). The diagnosis was based on Sepsis 3.0 definition. There was no age restriction (except for neonates). The exclusion criteria were as followed: missing date, malignant tumor and immunocompromised state (e.g., long-term use of glucocorticoids, immunosuppressants). To check whether our sample size was large enough to develop a clinical prediction model, we applied the approach proposed by Riley et al. [12]. According to a 28-day mortality of 30% [13] and 5 candidate predictors, at least 323 patients were required. Eventually, 349 patients were included in the study. The survival time of each patient was recorded. If the patient survived more than 28 days, then the length of hospital stay or the time of transfer was recorded.

All the patients included in the study had received antibiotics and then transferred to ICU department. The patients were treated with standard therapeutic strategies under the instructions of Surviving Sepsis Campaign Guideline [1]. All the laboratory tests (including nCD64 index) were routinely performed in our institution for the diagnosis and assessment of disease progression. The characteristics of the study population were summarized in Table 1.

### Data extraction

Data were retrieved from electronic medical record system in our hospital. Demographic characteristics, histories, comorbidities, site of infection, admission laboratory results, APACHE II and SOFA score were collected. Blood indexes included white blood cells (WBC), neutrophils (NEU), lymphocytes (LYM), platelets (PLT), CRP, PCT, nCD64 index.

### Laboratory measurements

Blood samples were drawn from venous puncture right after presentation to ICU and then sent to laboratory department for analysis. CRP levels were quantified by IMMAGE Immunochemistry System (Beckman Coulter, Inc., CA, USA) using nephelometry test. PCT levels were analyzed by serum electrochemiluminescent immunoassay using the mini-VIDAS system (Biomerieux SA, France). The CD64 index was measured using the Cytomics FC 500 MPL (Beckman Coulter, Inc., CA, USA). Specifically, 50 µL of EDTA anticoagulated whole blood was collected and 5 µL CD64-FITC, 5 µL CD33-PE, 5 µL CD45-ECD (Beckman Coulter, Inc., CA, USA) antibody were added to the sample. The sample was then mixed thoroughly and incubated at room temperature in the dark for 15 min. Erythrocyte lysin was then added and mixed and incubated at room temperature in the dark for 10 min. Finally, 500 µL PBS was added. Monocytes, neutrophils and lymphocytes were obtained by CD45/CD33-gating, and the median fluorescence intensity (MFI) of CD64 on the cells was analyzed. nCD64 index = (nCD64 MFI/lymCD64 MFI)/(mCD64 MFI/nCD64 MFI). All the tests were professionally performed by laboratory technicians according to manufacturer’s instructions.

### Statistical analysis

Data were expressed as median and interquartile range (IQR) or number and percentage. Groups were compared using chi-square test for categorical variables and Mann–Whitney U test for continuous variables. Cox regression model was employed to identify the potential biomarkers for predicting 28-day mortality of sepsis. Hazard ratio (HR) with 95% confidence interval (CI) was utilized for both univariate analysis and multivariate Cox regression model. Age, sex, comorbidities were adjusted for model 1 and those factors plus site of infection, lymphocytes, platelets, APACHE II and SOFA score were adjusted for model 2.

Receiver operating characteristic (ROC) curves was performed to evaluate the predictive accuracy of CRP, PCT, nCD64 index, nCD64 index plus PCT, nCD64 index plus CRP, by comparing the area under curves (AUC). We compared the AUC values among different biomarkers using the method of Hanley and McNeil [14]. Net reclassification improvement (NRI) and the integrated discrimination improvement (IDI) with 95% CI were used to measure the studied models` predictive performance. The optimal cut-off values, sensitivity, specificity, Youden index, positive predictive value (PPV), and negative predictive value (NPV) for each parameter were calculated in predicting the 28-day mortality in septic patients.

All patients were divided into four groups (from 0 to 3) based on the frequency of optimal cut-off values, and each group was compared according to the 28-day mortality using Kaplan–Meier survival curves and HR (with 95% CI). SPSS 23.0 (version 22.0, Chicago, USA), MedCalc Software (version 19.0, MedCalc Software, Mariakerke, Belgium) were used. A two-sided P value < 0.05 was considered statistically significant.

## Results

### Patient demographics

A total of 349 septic patients were enrolled and 250 (71.6%) of them survived more than 28 days. There were no significant differences regarding sex, age, smoking, drinking and comorbidities between the survivors and non-survivors (P > 0.05). However, there were significant differences in terms of infection distribution between the two groups. Non-survivors suffered more from infection of enterocoelia, skin and soft tissue, urinary system and central nervous system than survivors (P < 0.05). Generally, the deceased had lower lymphocyte, platelet counts, but much higher CRP, PCT and nCD64 index levels. Also, non-survivors suffered more from sepsis indicated by APACHE II and SOFA score (Table 1).

**Table 1:** Characteristics of the study population

Variables	Overall (n = 349)	Survivor (n = 250)	Non-survivor (n = 99)	p
Demographics				
Age, years	66 (52–77)	65.5 (50–77)	66 (55–75)	0.527
Males/females, n (%)	229/120 (65.6/34.4)	167/83 (66.8/33.2)	62/37 (62.6/37.4)	0.459
History				
Smoking, n (%)	94 (26.9)	69 (27.6)	25 (25.3)	0.656
Drinking, n (%)	33 (9.5)	25 (10)	8 (8.1)	0.581
Comorbidities				
Hypertension, n (%)	172 (49.3)	126 (50.4)	46 (46.5)	0.507
DM, n (%)	107 (30.7)	75 (30)	32 (32.3)	0.671
CHD, n (%)	53 (15.2)	38 (15.2)	15 (15.2)	0.991
CRF, n (%)	38 (10.9)	31 (12.4)	7 (7.1)	0.15
CHF, n (%)	38 (10.9)	31 (12.4)	7 (7.1)	0.15
COPD, n (%)	23 (6.6)	15 (6)	8 (8.1)	0.48
CVD, n (%)	46 (13.2)	34 (13.6)	12 (12.1)	0.713
Site of infection				
Lower respiratory tract, n (%)	246 (70.5)	201 (80.4)	45 (45.5)	< 0.001
Enterocoelia, n (%)	52 (14.9)	18 (7.2)	34 (34.3)	< 0.001
Skin and soft tissue, n (%)	40 (11.5)	16 (6.4)	24 (24.2)	< 0.001
Urinary system, n (%)	82 (23.5)	40 (16.0)	42 (42.4)	< 0.001
Central nervous system, n (%)	24 (6.9)	12 (4.8)	12 (12.1)	0.015
Unknown, n (%)	21 (6.0)	13 (5.2)	8 (8.1)	0.308
Blood parameters				
WBC (× 10^9^/L), median [IQR]	13.06 (9.41–18.12)	12.82 (9.56–17.32)	14.33 (8.29–20.45)	0.446
NEU (× 10^9^/L), median [IQR]	11.57 (7.76–16.11)	11.12 (7.88–15.34)	12.84 (7.44–18.72)	0.186
LYM (× 10^9^/L), median [IQR]	0.92 (0.55–1.45)	1.01 (0.63–1.55)	0.68 (0.31–1.08)	< 0.001
PLT (× 10^9^/L), median [IQR]	197 (129–243)	206 (140–248.25)	160 (91–223)	0.001
CRP(mg/dL), median [IQR]	75.1 (42.10–123.5)	61.7 (35.68–84.65)	149 (84.2–234.00)	< 0.001
PCT(ng/mL), median [IQR]	4.77 (1.16–19.14)	2.70 (0.86–6.31)	28.87 (8.37–99.40)	< 0.001
nCD64 index, median [IQR]	1.39 (0.61–3.10)	1.11 (0.45–1.62)	4.76 (2.57–7.30)	< 0.001
Severity scores				
APACHE II	20 (14.5–26)	16 (13–23)	30 (25–35)	< 0.001
SOFA	8 (5–11)	6 (4–9)	13 (10–15)	< 0.001

### Predictors of 28-day mortality in septic patients

As demonstrated in Table 2, in the univariable Cox proportional hazards model, CRP, PCT, nCD64 index were associated with 28-day mortality of sepsis (P < 0.001). However, in the multivariable Cox proportional hazards model 1 and model 2, CRP, nCD64 index were found to be associated with 28-day mortality (P < 0.05). After adjusting for age, sex, comorbidities, site of infection, lymphocytes, platelets, APACHE II and SOFA score, CRP and nCD64 index were considered as independent predictors of 28-day mortality in septic patients.

**Table 2:** Univariable and multivariable Cox regression models predicting 28-day mortality

Variables	Univariable model		Multivariable model 1		Multivariable model 2	
	h (95% CI)	p	HR (95% CI)	p	HR (95% CI)	p
CRP	1.008 (1.006–1.009)	< 0.001	1.006 (1.005–1.008)	< 0.001	1.004 (1.002–1.006)	< 0.001
PCT	1.002 (1.001–1.003)	< 0.001	–	–	–	–
nCD64	1.412 (1.345–1.482)	< 0.001	1.393 (1.319–1.471)	< 0.001	1.263 (1.187–1.345)	< 0.001

### The predictive efficacy of 28-day mortality for the studied parameters

**Fig. 2:**
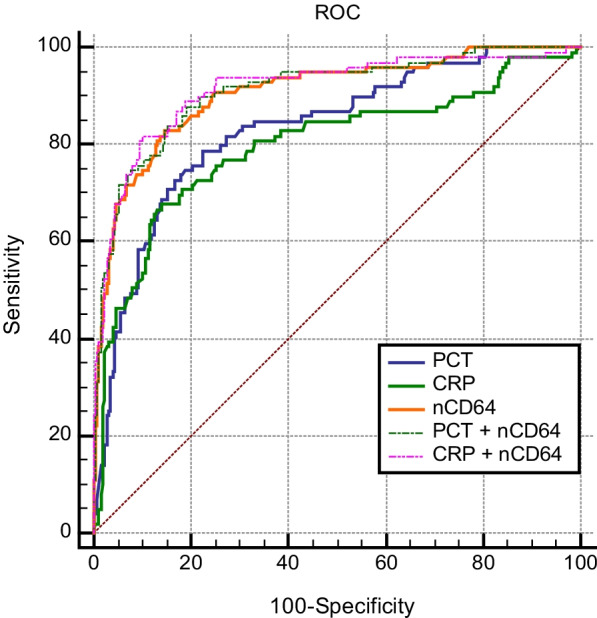
ROC analysis of the studied biomarkers for predicting 28-day mortality in sepsis

AUC was used to discriminate the predictive efficacy of 28-day mortality. The AUC values of CRP, PCT, nCD64 index, nCD64 index plus PCT, nCD64 index plus CRP, were 0.798, 0.833, 0.906, 0.910, 0.916, respectively (Fig. 2; Table 3). CRP plus nCD64 index presented the largest AUC value (but not superior to nCD64 index, PCT plus nCD64 index, with p value 0.265, 0.548, respectively).

Next, NRI and IDI analysis were performed to assess the risk prediction model performance. nCD64 index plus PCT and nCD64 index plus CRP showed higher NRI than CRP. nCD64 index, nCD64 index plus PCT and nCD64 index plus CRP showed higher IDI than CRP. Notably, nCD64 index combined with CRP could better reclassify patients into a more appropriate 28-day mortality risk category.

The sensitivity, specificity, cut-off point, PPV, NPV and Youden index were calculated to evaluate the predictive efficacy of the biomarkers comprehensively (Seen in Table 4). PCT plus nCD64 showed the highest sensitivity (87.9%) and NPV (94.4%). CRP + nCD64 showed the highest specificity (90.4%) and PPV (77.1%). The cut-off values were listed in Table 4.

Collectively, nCD64 plus CRP performed best in prediction, discrimination, and reclassification of the 28-day mortality risk in sepsis.

**Table 3:** The discriminating capability of biomarkers in predicting 28-day mortality of septic patients

Variables	CRP	PCT	nCD64	PCT + nCD64	CRP + nCD64
AUC	0.798 (0.752 to 0.839) NA	0.833 (0.790 to 0.871) P = 0.289	0.906 (0.870 to 0.935) P = 0.001	0.910 (0.875 to 0.938) P < 0.001	0.916 (0.881 to 0.943) P < 0.001
NRI	NA	− 0.953 (− 1.154 to − 0.157) P = 0.010	0.182 (− 0.068 to 0.470) P = 0.143	0.572 (0.031 to 1.102) P = 0.038	1.137 (0.936 to 1.335) P < 0.001
IDI	NA	− 0.134 (− 0.204 to 0.052) P < 0.001	0.135 (0.048 to 0.226) P < 0.001	0.136 (0.044 to 0.224) P = 0.01	0.232 (0.157 to 0.306) P < 0.001

**Table 4:** Comparison of the sensitivity and specificity of the studied biomarkers in predicting 28-day mortality

Variables	Sensitivity (%)	Specificity (%)	Cut-off point	PPV (%)	NPV (%)	Youden index	
CRP	67.7	86	105.4 mg/dL	65.7	87	0.537	
PCT	78.8	77.6	7.67 ng/mL	58.2	90.2	0.564	
nCD64	81.8	86.4	2.265	70.4	92.3	0.682	
PCT + nCD64	87.9	80.8	NA	64.4	94.4	0.687	
CRP + nCD64	81.8	90.4	NA	77.1	92.6	0.722	

### Multi-marker approach predicting 28-day mortality for sepsis

**Fig. 3:**
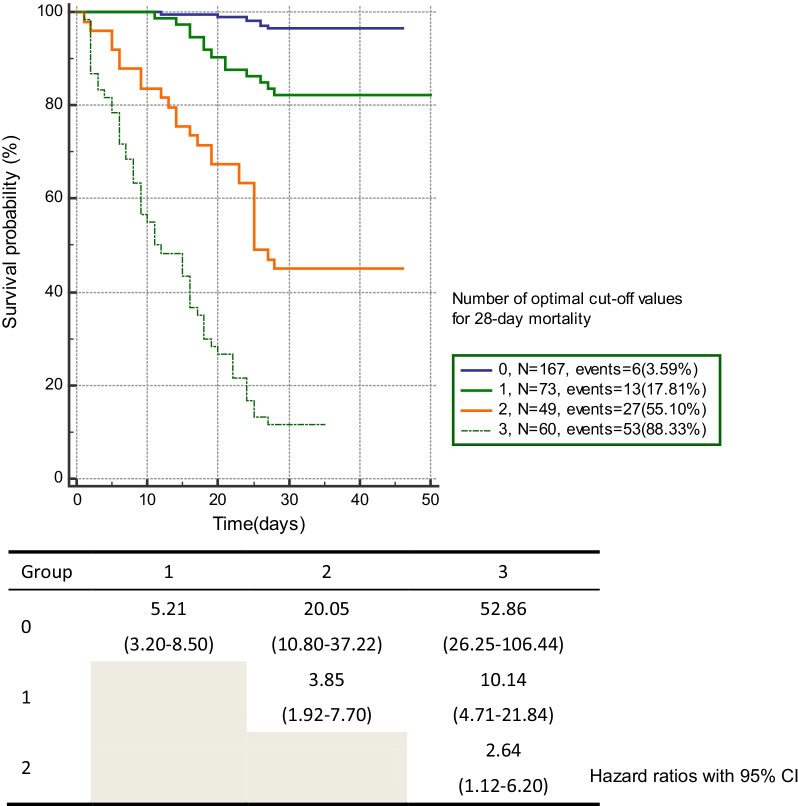
Multi-marker approach predicting 28-day mortality for sepsis

We employed a multi-marker approach using the number of data exceeding the cut-off point for each biomarker to predict 28-mortality for septic patients. Mortality rate in each group showed a stepwise increase: 3.59% in Group 0, 17.81% in Group 1, 55.10% in Group 2, 88.33% in Group 3. Group 3 showed higher HR compared with Group 0, 1, 2; 52.86 (95% CI 26.25–106.44), 10.14 (95% CI 4.71–21.84), 2.64 (95% CI 1.12–6.20) (Fig. 3).

## Discussion

In the present study, we have shown that nCD64 index was a promising prognostic biomarker for 28-day mortality in ICU septic patients, with AUC 0.906, superior to CRP, PCT. Additionally, the combination of CRP plus nCD64 index showed better predictive efficacy demonstrated by ROC, NRI and IDI analysis. Multi-marker approach, by dividing patients into different groups based on the frequency of the optimal cut-off values, showed stepwise increased 28-day mortality.

CRP has been studied for its prognostic efficacy for sepsis. But the results intriguingly differ. Several studies compared the CRP levels in ICU survivors vs. non-survivors with sepsis without finding any differences [13, 15–17]. The sample size of the four studies was smaller than that of a recent research. Hazem Koozi et al. [7] studied 851 septic patients and found that an admission CRP level > 100 mg/L was associated with 30-day mortality. Additionally, a prospective study of 313 patients showed that ICU patients with higher CRP levels on admission had higher mortality and risk of organ failure [18]. Another study including 576 ICU patients also found that CRP predicted ICU mortality independent of APACHE II score system [19]. Our study was in line with these results. We found CRP was an independent predictive factor of sepsis for short term mortality. CRP showed a decent prognostic value, with AUC 0.798 (95% 0.752–0.839) and cut-off value 105.4 mg/dL.

Typically, PCT is a more reliable marker of sepsis than CRP, but not all studies support that. A recent study showed that serum PCT level on emergency admissions could not predict 28-day mortality for septic patients [20]. Also, CRP was a better marker than PCT for sepsis induced by respiratory infection [21, 22]. This coheres with our clinical experience. Respiratory associated septic patients usually present relatively low PCT level, while gastrointestinal, skin and soft tissue associated septic patients present high PCT level. In our study, 70.5% of the included patients had lower respiratory infection, and the septic population may affect the result. Additionally, PCT is often used as a biomarker to distinguish the presence of bacterial infection [23]. However, in the present study, we included septic patients with different kinds of pathogens, which might influence the results.

A study including 47 septic patients found that nCD64 was associated with severity of sepsis and organ failure [24]. Also, another study including 797 ICU patients found that nCD64 predicted ICU mortality [8]. The results were similar to ours. Although the literature on the prognostic utility of nCD64 is not extensive, it remains a promising candidate.

In the light of the fact that sepsis is a highly complex immunological syndrome, involving simultaneous implication of both hyperinflammation and immunosuppression, a single biomarker will never be sufficient to gauge a patient`s overall immune status and combination of different markers would be better than single biomarker assessment. Our results are novel with respect to combined use of CRP, PCT, nCD64 index as markers of sepsis. As the number of data exceeding cut-off values increased from 0 to 3, the 28-day mortality increased in a stepwise pattern.

Improvement in survival depends on early recognition, acute stratification and the ensuing treatment, as a consequence of which, identifying individual with high risk has become a well-recognized priority [1, 3]. Prognostic biomarkers may be useful to triage patients in special environments, such as in the emergency room, when the information provided can help clinicians to decide whether hospitalization is necessary and, if so, on the ICU or on the regular floor. Also, risk stratification and prognostication help to identify patients who are at higher risk and then may benefit from extensive treatment beyond the standard therapy. By evaluating the early inflammatory-immune status of septic patient, clinicians could optimize the treatments (especially immunomodulatory treatments for sepsis-induced immunosuppression), thus to reduce the mortality.

Several limitations also need to be addressed. First, a key limitation was that our work was a single-center retrospective study and the prognostic model was not validated by external dataset due to limited conditions. Furthermore, we only evaluated the predictive efficacy of the admission levels of CRP, PCT, nCD64 index, other than their changes over disease course. Thirdly, we only included nCD64 index due to limited condition, other than standardized assay. Standardized assay would facilitate generalizability and comparison with other cohorts using the same methodology.

## Conclusions

Compared with PCT, CRP, nCD64 index showed superior prognostic performances, and their combined use improved the predictive efficacy. Multi-marker approach using CRP, PCT, nCD64 index seems to be objective and useful for the prognosis prediction in septic patients.

## Data Availability

The datasets supporting the conclusions of this article will not been deposited publically, but are available from the corresponding author on reasonable request.

## Abbreviations

CRP: C-reactive protein
PCT: Procalcitonin
nCD64: Neutrophil CD64
ROC: Receiver-operating characteristic curve
HR: Hazard ratio
AUC: Area under the ROC curve
ICU: Intensive care unit
DM: Diabetes mellitus
CHD: Coronary heart disease
CRF: Chronic renal failure
CHF: Chronic heart failure
COPD: Chronic obstructive pulmonary disease
CVD: Cerebrovascular disease
WBC: White blood cells
NEU: Neutrophils
LYM: Lymphocytes
PLT: Platelets
IQR: Interquartile range
CI: Confidence interval
NIR: Net reclassification improvement
IDI: Integrated discrimination improvement
PPV: Positive predictive value
NPV: Negative predictive value
